# Selective impairment of attentional set shifting in adults with ADHD

**DOI:** 10.1186/s12993-018-0150-y

**Published:** 2018-11-10

**Authors:** Aquiles Luna-Rodriguez, Mike Wendt, Julia Kerner auch Koerner, Caterina Gawrilow, Thomas Jacobsen

**Affiliations:** 1 Experimental Psychology Unit, Helmut-Schmidt-University/University of the Federal Armed Forces Hamburg, Holstenhofweg 85, 22043 Hamburg, Germany; 2 Faculty of Human Sciences, Medical School Hamburg, Hamburg, Germany; 3 Educational Psychology, Helmut-Schmidt-University/University of the Federal Armed Forces Hamburg, Hamburg, Germany; 4 School Psychology, Eberhard Karls Universität Tübingen, Tübingen, Germany; 5 Center for Individual Development and Adaptive Education of Children at Risk (IDeA), Frankfurt, Germany

**Keywords:** ADHD, Attentional set, Task switching, Cognitive control, Executive function

## Abstract

**Background:**

Task switch protocols are frequently used in the assessment of cognitive control, both in clinical and non-clinical populations. These protocols frequently confound task switch and attentional set shift. The current study investigated the ability of adult ADHD patients to shift attentional set in the context of switching tasks.

**Method:**

We tested 38 adults with ADHD and 39 control adults with an extensive diagnostic battery and a task switch protocol without proactive interference. The experiment combined orthogonally task-switch vs. repetition, and attentional set shift vs. no shift. Each experimental stimulus had global and local features (Hierarchical/“Navon” stimuli), associated with corresponding attentional sets.

**Results:**

ADHD patients were slower than controls in task switch trials with a simultaneous shift of attention between global/local attentional sets. This also correlated significantly with diagnostic scales for ADHD symptoms. The patients had more variable reaction times, but when the attentional set was kept constant neither were they significantly slower nor showed higher task switch costs.

**Conclusion:**

ADHD is associated with a deficit in flexible deployment of attention to varying sources of stimulus information.

## Background

Daily life difficulties experienced by individuals suffering under ADHD symptoms have frequently been linked to deficits in executive functions, a class of mental processes assumed to organize cognitive activity in the service of goal-directed behavior (e.g., [1–8]). Although the concept of executive functions is not well defined, a core aspect thereof relates to the ability to adjust mental sets according to changing task requirements and context conditions. A prevalent means of investigating such adjustment is the task switching (TS) paradigm (overview in [9, 10]). In standard task switching studies, participants execute two different tasks, usually involving the same set of stimuli, in varying sequences. Response performance is typically worse in task switch trials (i.e., trials preceded by a trial associated with the other task) than in task repetition trials (i.e., trials preceded by a trial associated with the same task). Although these *task switch costs* have been attributed to executive processes of task-set reconfiguration, they may also be accounted for in terms of stimulus- and task-specific proactive interference.

ADHD-related impairment in conditions associated with TS has been reported in several studies (e.g., [11–13]). In light of the fact that a deficit in TS performance may arise from a multitude of processes involved in task switching, the current study examined evidence for ADHD-related impairment concerning a particular component of task-set reconfiguration. In many TS experiments the tasks between which participants switch differ with regard to the relevant perceptual features of the stimulus. Participants may switch between color and shape identification tasks [14, 15], between reporting the number of –identical- stimulus elements vs. their identity [11, 12, 16], or between classifying digits vs. letters [13, 17]. TS in these situations may involve a shift of the attentional set (AtS), that is, reconfiguring attentional weights assigned to relevant and irrelevant stimulus features. This contrasts with conditions in which two different tasks have to be applied to the same perceptual attributes of the stimulus. For instance, [18] asked children with and without ADHD to switch between classifying a digit stimulus as either odd/even or smaller/larger than 5, and failed to find a difference in TS costs. Crucially, in the former case TS performance may be supported by deploying attention to the perceptually distinct attributes of the stimulus that define the target information of the current task (see [19] for a demonstration of dissociable attentional sets in the domain of spatial stimulus selection). Enhancement of switch costs for individuals with ADHD might thus go back to a deficit in flexible adjustment of attention, that is, in efficient re-weighting of attentional weights assigned to changing perceptual attributes.

Findings obtained in a recent Eriksen Flanker Task study [20] involving a manipulation of the ratio of congruent and incongruent trials, are in line with the notion of a deficit in flexible adjustment of attention in patients with ADHD. Specifically, whereas the control group showed a higher congruency effect in blocks with 80% congruent stimuli than in blocks with 20% congruent, in the ADHD group, the congruency effect was low regardless of the congruent/incongruent ratio. This suggests that control participants adjusted their attention depending on distractor utility (cf. [21], overview in [22]), while the ADHD group appeared to maintain a strong attentional focus regardless of context conditions.

Because in selective attention tasks, such as Eriksen Flanker, the target stimulus contains all information needed for successful task performance, these tasks can be accomplished by maintaining a strongly focused state of attention regardless of contextual changes. Deficits in flexible attentional adjustment should be associated with more detrimental consequences, however, if task-relevant stimulus information must be extracted from frequently changing stimulus features. As noted above, this is the case in TS studies which involve tasks associated with different target stimulus features. In light of the fact that tasks combined in TS experiments are associated with a multitude of additional processing differences (i.e., cognitive operations of stimulus classification and response selection), however, it is conceivable that TS performance can be accomplished by relying on biasing processing independently of stimulus perception (e.g., [23]).

Standard TS protocols may thus not constitute an optimal means to assess attentional adjustment. To investigate a possible impairment in adjusting the set of stimulus selection, we followed the approach of [21]. This study used hierarchical stimuli [24], either big letters made out of small letters or big numbers made out of small numbers (see “Apparatus and stimuli”). The tasks were digit and letter identification, and each could be performed with two AtS levels, either global or local (big and small, respectively). With this method, task switch costs could be compared in conditions with and without the need to shift the AtS. In addition, congruency effects, exerted by the irrelevant stimulus level, served as indication for the degree of processing the irrelevant level. Task switch costs and congruency effects were larger when switching between tasks was associated with shifting attention between stimulus levels (mixed levels condition) than when target levels were kept constant (constant levels condition), suggesting a specific cost of shifting the set of stimulus selection as well as a lower degree of shielding performance against interference from information presented on the irrelevant level after a level switch.

Assuming that ADHD is associated with a particular deficit in flexible adoption of (task-specific) sets of stimulus selection one would predict particular enhancement of switch costs and congruency effects in the mixed levels condition for patients with ADHD compared to controls. By contrast, switching between the same tasks should not be particularly impaired when target levels are kept constant.

Interpretation of task switch costs in terms of executive functioning is usually rendered difficult by stimulus-specific effects of interference between tasks. TS performance seems particularly impaired if a stimulus is processed that was previously presented in the context of the other task (e.g., [25]), possibly reflecting stimulus-based cuing of task-set conflict rather than impaired cognitive reconfiguration. Unlike standard TS protocols that involve frequent occurrences of all stimuli in the context of both tasks, the procedure used in the current study avoids such proactive interference by presenting qualitatively different stimuli in the two tasks (i.e., global digits made up of local digits vs. global letters made up of local letters [21]).

## Methods

### Participants

Demographic data of our sample is displayed in Table 1. Thirty-eight adults (*M* age = 36.14 years, *SD *= 12.17; 17 women) with a diagnosis of ADHD as their primary disorder, were recruited in a Hamburg neurological outpatient practice which is specialized in the diagnosis and treatment of ADHD in adults. During a regular appointment in the practice patients were informed about the possibility to participate in the experiment right away or make an appointment. Patients were included into the sample if they had received a clinical diagnosis of ADHD. Fourteen patients received their diagnoses before the age of 18 years. Twelve patients reported to have comorbid diagnoses. Thirty-three patients were treated with extended-release methylphenidate, two with Serotonin–Noradrenalin-Reuptake-Inhibitors and four were not taking medication. Patients were instructed to take their medication as usual and during the experiment 26 patients were under the influence of their medication (i.e., had taken extended-release Methylphenidate within 12 h before the experiment or Serotonin–Noradrenalin-Reuptake-Inhibitors in the last-2 weeks). Twenty-four patients had received or were receiving psychotherapy.

**Table 1:** Demographic description of the sample by group

	ADHD group (*n* = 38)	Control group (*n* = 39)	Group difference
Gender (*n* and % of women)	17 (44.7%)	19 (48.7%)	0.903^a^
*M*-age (*SD*)	36.14 (12.71)	33.61 (9.81)	0.330^b^
Country of birth Germany	35 (92.1%)	32 (82.1%)	0.331^a^
Highest educational level			0.947^a^
Primary school (Grundschule)	1 (2.6%)	0 (0.0%)	
Secondary school (Haupt- and Realschule)	10 (26.3%)	10 (25.6%)	
Secondary school and vocational training (Haupt-, Realschule mit Berufsabschluss, Gymnasium)	3 (7.9%)	4 (10.3%)	
University entrance degree (Fachhochschulreife, Abitur)	13 (34.2%)	13 (33.3%)	
University degree (Hochschulabschluss, Fachhochschulabschluss)	10 (26.4%)	11 (28.2%)	
Ph.D. (promotion)	1 (2.6%)	1 (2.6%)	
Other psychological diagnoses (%)	12 (31.6%)	2 (5.1%)	0.007^a^
Psychotherapy	24 (63.2%)	4 (10.3%)	< 0.001^a^

^a^χ^2^, ^b^ *p*-value of *t* test

The adults without ADHD of the control group were recruited using various strategies. We asked adults without any disorders that were accompanying patients into the practice to participate, we posted calls for participants on a website for job advertisements and addressed people waiting in local employment offices to recruit a diverse sample comparable to the sample with ADHD. Participants in the control group were excluded if they received clinically relevant (*t*-score above 65, t-scores have a mean of 50 and a standard deviation of 10) scores in two or three symptom scales of the CAARS inattention/memory problems, hyperactivity/restlessness and impulsivity/emotional lability. Two participants were excluded due to this criterion. The final control group included 39 control adults (*M* age = 33.61 years, *SD* = 9.81, 19 women). Two participants reported to have received psychological diagnoses in the past (depression and adjustment disorder) and four reported psychotherapy. We compared the group with ADHD with the group without ADHD with regards to gender, age, country of birth, degree of education and did not find any significant group differences (see Table 1). As expected, patients reported more comorbid diagnoses and more psychological treatment.

The experiment and the diagnostic procedure were performed by trained student assistants in a quiet room of the neurological outpatient practice. The window blinds were closed to avoid distraction and ensure the same lighting conditions for all participants. After giving informed consent the participants were briefly interviewed about their demographics and ADHD diagnosis before they performed in the experiment. Subsequently, participants were asked to fill out the questionnaires (Conners’ Adult ADHD Rating Scales [CAARS], Brief-Symptom-Inventory [BSI], Self-Control Scale [SCS-K-D]) as well as to take part in an IQ screening (Wechsler Adult Intelligence Scale fourth edition [WAIS-IV]) and in the tests of attention (test battery for attentional performance [TAP]). Finally, the last part of the session was the clinical interview (Wender-Reimherr-Interview [WRI]). After completion, participants received their reimbursement and were informed about the possibility to receive a written report about the results of the study in general. The session took approximately 2 h.

### Diagnostic measures

The following table displays the results of the diagnostic measures. To avoid habituation or practice effects diagnostic measured used in this study differed from the diagnostic measures usually applied in the neurological outpatient practice where we recruited participants.

#### Conners’ Adult ADHD Rating Scales (CAARS)

The CAARS is a clinical questionnaire assessing attention problems in adults [26]. We used the German version [27]. The scales inattention/memory problems, hyperactivity/restlessness, impulsivity/emotional lability and problems with self-concept assess the current symptoms. Furthermore, there are three scales assessing the DSM-IV symptoms of inattention and hyperactivity/impulsivity and an ADHD index. We used the long self-report version with 66 items. The scales show high internal consistency (Cronbachs Alpha > 0.85) and an average test–retest reliability of 0.88 [27]. Groups differed highly significant in all scales of the CAARS (Table 2).

**Table 2:** Diagnostic data of the sample by group

	ADHD group (*n* = 38)	Control group (*n* = 39)	Group difference
CAARS			
Inattention/memory problems (t-value)	70,63 (16,26)	44.21 (8.09)	< 0.001
Hyperactivity/restlessness (t-value)	66.24 (14.38)	48.69 (8.80)	< 0.001
Impulsivity/emotional lability (t-value)	67.03 (17.31)	44.64 (6.50)	< 0.001
Problems with self-concept	10.24 (5.01)	4.10 (2.57)	< 0.001
DSM: inattention	15.05 (6.11)	3.87 (2.77)	< 0.001
DSM: hyperactivity/impulsivity	12.11 (6.13)	4.36 (3.33)	< 0.001
ADHD index	19.66(7.60)	6.74 (3.56)	< 0.001
WRI			
Inattention	7.53 (2.90)	1.41 (1.65)	< 0.001
Hyperactivity	3.11 (2.08)	0.69 (1.15)	< 0.001
Hot temper	3.87 (2.23)	1.00 (1.43)	< 0.001
Mood instability	5.05 (2.68)	1.56 (1.68)	< 0.001
Over reactivity	2.68 (2.11)	0.69 (1.26)	< 0.001
Disorganization	5.61 (3.36)	1.72 (1.69)	< 0.001
Impulsivity	5.26 (2.87)	2.79 (2.35)	< 0.001
WRI global score	11.55 (5.28)	3.13 (3.11)	< 0.001
TAP—working memory			
Correct hits	11.63 (3.95)	13.38 (1.89)	0.017
Errors of omission	3.95 (0.64)	1.89 (0.30)	0.017
BSI			
Somatization	0.63 (0.69)	0.23 (0.42)	0.004
Obsessive–compulsive	1.65 (0.97)	0.45 (0.48)	< 0.001
Interpersonal sensitivity	1.36 (1.14)	0.38 (0.45)	< 0.001
Depression	1.27 (1.06)	0.22 (0.29)	< 0.001
Anxiety	1.03 (0.81)	0.32 (0.37)	< 0.001
Hostility	1.18 (0.90)	0.25 (0.25)	< 0.001
Phobic anxiety	0.64 (0.95)	0.15 (0.19)	0.003
Paranoid ideation	1.18 (1.00)	0.31 (0.38)	< 0.001
Psychoticism	0.96 (0.94)	0.21 (0.29)	< 0.001
SCS-K-D	38.89 (3.33)	38.54 (2.82)	0.614
WAIS-IV matrices (raw score)	18.47 (4.95)	18.79 (3.81)	0.750
WAIS-IV vocabulary (raw score)	36.92 (11.97)	37.15 (10.83)	0.929
WAIS-IV estimated IQ	91.97 (15.33)	92.15 (11.29)	0.953

*CAARS* Conners’ Adult ADHD Rating Scales, *WRI* Wender-Reimherr-Interview, *TAP* test battery for attentional performance, *BSI* brief-symptom-inventory, *SCS-K-D* Self-Control Scale, *WAIS-IV* Wechsler Adult Intelligence Scale fourth edition

#### Wender-Reimherr-Interview (WRI)

The WRI has been published in German as part of the Homburger ADHD-Scales for adults test battery (HASE) [28]. The WRI is based on the American WRI [29]. In a structured interview psychopathological items are rated by the interviewer on a scale from 0 (*not present*) to 2 (*medium or high*). The 28 items are part of seven subscales: inattention, hyperactivity, hot temper, mood instability, over reactivity, disorganization, impulsivity. The score is a sum of all items. Furthermore, there is a global rating for each scale judging symptom on a scale from 0 (*not present*) to 4 (*severe*). The WRI Global Score is a sum of the seven global ratings. Groups differed significantly in all scales and the global score of the WRI (Table 2). The interrater reliability for diagnoses of ADHD is kappa = 1.0; ICC = 0.92. The reliability for the total score is α = 0.82 [28].

#### Test battery for attentional performance (TAP)

The subtest working memory from the TAP 2.3 [30] was administered. This working memory 2-back task, asked participants to press a button whenever the one-digit-number appeared on the screen was the same as the one before the last one. Patients had less correct hits and more errors of omission (Table 2). The split-half reliability for working memory was determined on the basis of odd–even splits with *r* being 0.847 for median reaction time, 0.885 for errors and 0.742 for omissions [30].

#### Brief-symptom-inventory (BSI)

The BSI [31] provides an overview of self-reported clinically relevant psychological symptoms in adolescents and adults. The BSI is the short version of the SCL-R-90 [32], which measures the same dimensions. Items for each dimension of the BSI were selected based on a factor analysis of the SCL-R-90, with the highest loading items on each dimension selected for the BSI [33, 34]. The BSI requires only 8–10 min to complete and consists of 53 items covering nine symptom dimensions: somatization, obsessive–compulsive, interpersonal sensitivity, depression, anxiety, hostility, phobic anxiety, paranoid ideation and psychoticism. The BSI has internal consistencies from α = 0.63 to α = 0.85 and retest-reliabilities from *r* = 0.73 to *r *= 0.92. Groups differed significantly in all scales of the BSI (Table 2).

#### Self-Control Scale (SCS-K-D)

The SCS-K the German adaption of the Brief Self-Control Scale (BSCS, [35]. The unidimensional questionnaire assesses self-control with 13 items which are rated on a 5-point, Likert scale with possible answers ranging from 1 (*not at all like me*) to 5 (*very much like me*). The internal consistency is α = 0.81. Patients and controls did not differ in self-control (Table 2).

#### Wechsler Adult Intelligence Scale fourth edition (WAIS-IV)

We used two subtests from the German version of the WAIS-IV [36]: Matrix reasoning and vocabulary. These two subtests have been shown to form a good indicator for general cognitive abilities testing in an economic way (*r* = 0.86 with the full test battery, [37]). The internal consistency of this two-test short form is high *r* = 0.94 [37]. A full IQ can be estimated by using these two subtests. The estimated full IQ in our sample was surprisingly low (Table 2), given the educational level. This could be due to the fact that in a complete administration of the WAIS-IV, three other subtests are administered before the matrix reasoning and vocabulary subtest. Therefore, participants could be less experienced with the format of the test if only the two subtests are administered [37]. However and most importantly, groups did not differ in the estimated IQ or in the raw scores of the two subtests used (Table 2).

### Reaction time experiment

#### Apparatus and stimuli

**Fig. 1:**
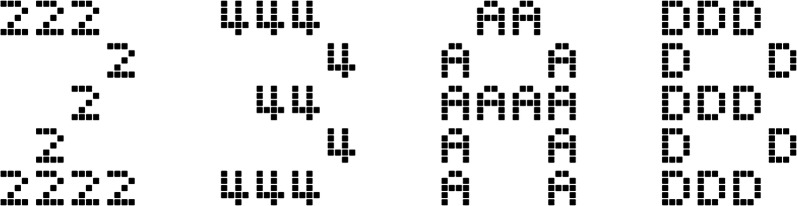
Examples of the stimuli used in the experiment. Left to right, the first and third stimuli are congruent in the global/local target levels, the second and fourth are incongruent

Stimuli were presented using a standard PC with a 23-inch LCD screen (1920 × 1080 pix., latency < 3 ms), viewed at a distance of approx. 55 cm. The experiment was implemented with MATLAB R2010a and Psychtoolbox 3.0. Responses were recorded with a 1-ms time resolution QWERTZ keyboard. The stimuli were composed of the characters 1, 2, 3, 4, A, B, C and D. Each of these characters could occur on the global and on the local level. The stimuli were adapted from the font Silkscreen, one of the smallest raster fonts. Each character consists of a 5 × 5 pixel matrix (no anti-aliasing) but the ones used here were all 4 × 5 pixels in size (except ‘1’ hich was 3 × 5 pix.). The local characters consisted of 4 × 5 matrices of black squares with white fringes. The global level used the local level matrices as “pixels”. The background was white. The local and global levels of each stimulus were both either digits or letters, never a mix of digit and letter. 25% of the stimuli were congruent across the global/local level (for example, a global letter ‘B’ made out of local letters ‘B’). Between both levels, the stimuli had an appearance of “self-similarity” and the size difference was as small as possible (Fig. 1). The stimuli were presented in the form of 217 × 301 pixel bitmap pictures (local characters 0.71° × 0.88° visually, global characters 3.4° × 5.2°); the rest of the screen was a dark grey background. Each participant had two tasks, “digit” and “letter”, presented in a random order with equal probability. The relevant level of the stimuli was mapped to the response keys ‘F’, ‘V’, ‘N’ and ‘J’ in the intuitive left-to-right fashion: 1, A: F; 2, B: V; 3, C: N, and 4, D: J. The response keys were pressed by the participant’s middle and index fingers of both hands. Half of the experimental blocks had a constant AtS level for both tasks, either global or local. The other half has a mixed AtS levels, either global-number/local-letter or local-number/global-letter. Balancing which constant set, which mixed sets and whether participants start with constant or mixed sets, resulted in eight different versions of the experiment.

#### Procedure

Each trial presented first a task cue (“Zahl” or “Buchst.”, German for “number” and “letter” respectively) and then the hierarchical stimulus. Each trial consisted of a 500 ms blank screen, 200 ms cue, and a 200 ms presentation of the stimulus. The trial ended when a response key was pressed. The participants performed three 30-items long practice blocks, first the number task, then the letter task, and finally both tasks mixed. This was followed by four 80-trials experimental blocks. After this, the AtS was changed from constant to mixed levels (or vice versa). Participants were accordingly instructed, and performed another four experimental blocks (without previous practice). During the whole experiment, a 900 ms feedback was given for each false response, and after each experimental block the mean reaction time and error percentage was shown.

## Results

The first three trials of each block, trials with an incorrect response and trials immediately following these were excluded from analysis. Response times were aggregated according to the within-subject factors constant vs. mixed level, task switch vs. repetition and congruent vs. incongruent stimulus. Unless otherwise noted, all the following statistics were based on repeated-measures analysis of variance (ANOVAs) with two-tailed significance values. ANOVAs were conducted on the mean reaction times of correct responses and on the average error proportions. In the reaction time analysis, the three main within-subjects factors were highly significant. Constant level was faster than mixed level, *F*(1,75) = 67,236, p < 0.001, η_p_^2^ = 0.473. Task repetition was faster than task switch, *F*(1,75) = 134,350, p < 0.001, η_p_^2^ = 0.642. Congruent stimuli were responded to faster than incongruent ones, *F*(1,75) = 30.127, p < 0.001, η_p_^2^ = 0.287. Averaged over the within-subject factors, patients with ADHD were slower than controls, *F*(1,75) = 4.516, p < 0.037, η_p_^2^ = 0.057.

**Fig. 2:**
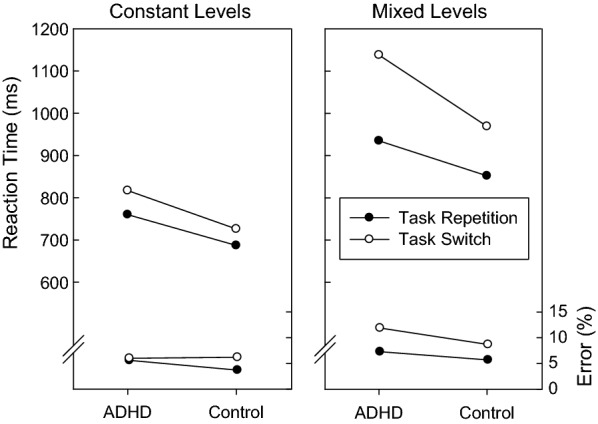
Mean reaction times and mean error percentages as a function of group (patients with ADHD, controls), target levels (constant, mixed), and task sequence (repetition, switch)

The only within-subjects factor that interacted significantly with the between-subjects factor ADHD/control was TS repetition/switch, *F*(1,75) = 134.350, p < 0.001, η_p_^2^ = 0.642; more interestingly, a three way interaction between constant/mixed target levels, task repetition/switch and ADHD/control was also significant, *F*(1,75) = 4.600, p < 0.035, η_p_^2^ = 0.058. Inspection of Fig. 2 shows that both groups of participants were associated with larger TS costs in the mixed target levels condition than in the constant target levels condition but this difference was more pronounced for the patient group (Table 3).

**Table 3:** Repeated measures ANOVA of mean reaction times

	F	p	η^2^ p
ADHD/control	4.516	0.037	0.057
Constant/mixed	67.236	< 0.001	0.473
Constant/mixed * ADHD/control	0.645	0.424	0.009
Repetition/switch	134.350	< 0.001	0.642
Repetition/switch * ADHD/control	8.232	0.005	0.099
Congruency	30.127	< 0.001	0.287
Congruency * ADHD/control	0.058	0.810	0.001
Constant/mixed * repetition/switch	51.464	< 0.001	0.407
Constant/mixed * repetition/switch * ADHD/control	4.600	0.035	0.058
Constant/mixed * congruency	12.647	0.001	0.144
Constant/mixed * congruency * ADHD/control	0.046	0.831	0.001
Repetition/switch * congruency	5.171	0.026	0.064
Repetition/switch * congruency * ADHD/control	0.090	0.764	0.001
Constant/mixed * repetition/switch * congruency	2.227	0.140	0.029
Constant/mixed * repetition/switch * congruency * ADHD/control	0.001	0.972	< 0.001

This interpretation was confirmed by conducting separate ANOVAs for the constant and the mixed levels conditions. The interaction between repetition/switch and ADHD/control was highly significant in the mixed levels case, *F*(1,75) = 11.846, p < 0.001, η_p_^2^ = 0.136, but not significant in the constant level case, *F*(1,75) = 0.595, p < 0.443, η_p_^2^ = 0.008. Due to the experiment’s design, it is conceivable that the presence of TS costs (without AtS shift) was an artifact. Half of the participants started with mixed levels, it is possible that in the following half of the experiment—unrequired—attention to the irrelevant AtS persisted. We examined therefore the constant level half of the experiment only in the participants that started with that condition (17 ADHD and 21 control participants). There was a trend towards TS costs for both groups, but no significant differences between ADHD patients and controls (*F*(1,36) = 1.463, p < 0.234, η_p_^2^ = 0.039).

We also examined the aggregated average error rate for each participant and factor combination (see Fig. 2). Again, all within-subjects main factors were highly significant and consistent with the reaction times [*F*(1,75) = 16.164, p < 0.001, η_p_^2^ = 0.177; *F*(1,75) = 44.573, p < 0.001, η_p_^2^ = 0.373; and *F*(1,75) = 58.379, p < 0.001, η_p_^2^ = 0.438, for constant/mixed target levels, task repetition/switch, and congruent/incongruent, respectively]. The interaction between ADHD/control and task repetition/switch was not significant, *F*(1,75) = 0.764, p < 0.385, η_p_^2^ = 0.010. The three-way interaction between ADHD/control, task repetition/switch, and constant/mixed target levels was not significant, *F*(1,75) = 2.262, p < 0.137, η_p_^2^ = 0.029. More importantly however, these last two interactions were numerically consistent with the reaction times, i.e., longer reaction times correspond to higher error rates. Separate ANOVAs for the constant levels condition and the mixed levels condition revealed that the interaction between repetition/switch and ADHD/control was significant in the constant levels *F*(1,75) = 4.325, p < 0.041, η_p_^2^ = 0.055, but not in the mixed levels, *F*(1,75) = 0.401, p < 0.528, η_p_^2^ = 0.005. As can be seen in Fig. 2, switch costs in the ADHD group were almost completely absent when the target level was kept constant.

A typical result found in the literature on executive control is that patients with ADHD show more reaction time variability. Although many studies report results in the form of standard deviations, this may be misleading because slower responses have higher numerical values, and tend to result in higher standard deviations. We opted instead to compute standard deviations for each participant and factor combination, and divided these values by the corresponding averages to obtain coefficients of variability, thus controlling the effect of speed differences; we then analyzed these coefficients with ANOVA using the same factors as in the reaction time analyses. Comparing ADHD patients with controls, the first show a significantly higher variability in response times, *F*(1,75) = 10.727, p < 0.001, η_p_^2^ = 0.125. The only other significant effect was that the mixed target levels condition had more variable response times than the fixed one, *F*(1,75) = 24.152, p < 0.001, η_p_^2^ = 0.244. No significant interaction with ADHD/control was found.

A final important question in this study is whether TS and AtS shift are related in general to ADHD deficits. We calculated a crude measure of performance in these executive functions simply by subtracting for each participant the average reaction time in task repetitions from the average in task switches (i.e., TS costs), and calculated this measure separately for the constant and mixed target levels halves of the experiment. Since these values are not corrected for age, sex or any other variable, we correlated these measurements with the raw values (no T-correction) of the scales attention, hyperactivity and impulsivity of the CAARS and WRI-HASE diagnostic tests. The results are displayed in Table 4.

**Table 4:** Pearson correlations between task switch-costs under constant/mixed target levels and diagnostic scales

	TS-cost constant		TS-cost mixed	
	r	Significance	r	Significance
CAARS inattention	0.282	0.0904	0.367	0.0113^a^
CAARS hyperactivity	0.329	0.0280^a^	0.253	0.1578
CAARS impulsivity	0.342	0.0211^a^	0.366	0.0113^a^
WRI inattention	0.222	0.2596	0.405	0.0031^b^
WRI hyperactivity	0.148	0.2701	0.172	0.2701
WRI impulsivity	0.220	0.2596	0.207	0.2596

Significance values are Holm–Bonferroni corrected for 12 comparisons, alpha 0.05

*TS* task switching, *AtS* attentional set, *CAARS* Conners’ Adult ADHD Rating Scales, *WRI* Wender-Reimherr-Interview

^a^Correlation is significant at the 0.05 level (two-tailed)

^b^Correlation is significant at the 0.01 level (two-tailed)

TS cost in the constant target level condition correlated significantly with the CAARS Hyperactivity and Impulsivity scales, mixed level TS correlated significantly with the CAARS inattention and impulsivity scales, but only the TS cost in the mixed condition had a significant correlation with the WRI-HASE attention scale. The diagnostic scales correlated among themselves in a much stronger way (not shown in the table, inattention r = 0.772, p < 0.01, hyperactivity r = 0.759, p < 0.01, impulsivity r = 0.566, p < 0.01.

## Discussion

Comparing the performance of persons with and without ADHD, patients were slower in task switch trials only when attention shifted to different stimulus features. When the attentional set was kept constant, task switch costs were present but not larger for patients than controls.

ADHD symptoms have long been assumed to be associated with a deficit in executive functioning, particularly with the flexible deployment of attention to varying sources of stimulus information. Findings of enhanced task switch costs may not be indicative of a deficit in AtS, however, because in standard task switching protocols, switches between tasks are often confounded with switches between perceptual features of the stimuli. Our experiment disentangled AtS from other components of task-set switch. The digit task had as targets 1, 2, 3 and 4, while the letter task used the letters A, B, C and D. Using stimuli with global and local features, half of the trial blocks had a constant target level (both tasks either local or global) while the other half required shifting the AtS on each task switch. The stimuli never combined digits and letters. In light of the heterogeneity of findings obtained in task switching studies involving patients with ADHD [11–13, 15, 18, 38, 39], lack of “pure” TS costs differences between patients with ADHD and controls has to be considered with caution. It must be emphasized that TS in the current study differed from usual TS protocols by the fact that the stimuli were strictly task-unique. Such conditions might not be associated with substantial demands of executive task-set reconfiguration. Also, this method avoided the occurrence of stimulus-related proactive interference, presumably a major source of task switch costs [25]. By consequence, task switch costs tended to be very small and may not be informative about the ability to shift task-sets or inhibit interference from the irrelevant task-set (cf. [21]).

Identifying deficits in executive functioning constitutes a major challenge in clinical (neuro)psychology. It has been criticized, in this connection, that commonly used procedures are characterized by lack of theoretical justification and more specific assessment of separable components of executive functions are desirable [40, 41] This seems to apply, particularly, to task switching performance as a diagnostic means, which has been found to be impaired in ADHD in some previous studies [8, 11, 15] but has been associated with comparably low effect sizes in meta-analyses [1, 42] Alongside with the search for discriminative subtypes in ADHD [43], isolating more specific components of task-set shift, as attempted in the current study with regard to shifting the attentional set, seems a valuable method to improve this situation, and allows a more detailed description of which cognitive processes may be affected.

The present study is consistent with the literature on ADHD showing more variable response times in TS experiments [44]. Also, we replicated previous findings on a student sample [21], demonstrating increased costs of switching tasks and increased congruency effects between global and local stimulus levels when task switching was associated with a shift of the attentional set.

However, patients did not display a larger difference in the size of the congruency effect in the mixed levels vs. the constant level condition than the control group. This result is reminiscent of previous findings of dissociations between overt responding to the global or local stimulus level and interference exerted by the other level (e.g., [45, 46]). Although we cannot rule out power problems, there is thus so far no indication of an impairment in shielding the processing of target stimulus information against distracting stimulus features. This pattern of findings is consistent with the observation in [20] that patients with ADHD showed no deficit regarding selective processing of target stimulus information but were reluctant to attenuate processing selectivity when the distractor stimuli were useful.

A strength of the current study is the representativeness of the sample and the well-matched control group. We had about an equal number of male and female patients [47, 48]. The educational level was very heterogeneous in both groups and the patients did not differ from controls with regards to IQ [49]. However, they did show high comorbid symptoms although they were being treated [50].

Concerning the relevance of our results for general ADHD symptoms, TS costs both in the constant and mixed target levels cases, correlated with the symptom scales of two ADHD diagnostic scales. Besides the unclear importance of TS without AtS shift, we deem our results robust enough to claim that attentional set shift is impaired in ADHD patients.

## Abbreviations

ADHD: attention deficit hyperactivity disorder
ANOVA: analysis of variance
AtS: attentional set
BSCS: Brief Self-Control Scale
BSI: brief symptom inventory
CAARS: Conners’ Adult ADHD Rating Scales
DSM-IV: diagnostic and statistical manual of mental disorders, revision IV
HASE: Homburger ADHS-Skalen für Erwachsene (German version of WRI)
SCS-K-D: Self-Control Scale
TAP: test battery for attentional performance
TS: task switch/task switching
WAIS-IV: Wechsler Adult Intelligence Scale, edition IV
WRI: Wender-Reimherr-Interview
